# Expectations of and willingness to pay for robotic‐assisted surgery: A prospective analysis before and after live presentation to the public

**DOI:** 10.1002/bco2.70304

**Published:** 2026-11-13

**Authors:** Christian Thomas, Mandy Ehrt, Benedikt Hoeh, Mario Hallaschek, Katharina Sonntag, Maximilian Winkelkotte, Emma Peklo, Martin Baunacke

**Affiliations:** ^1^ Department of Urology Medical Faculty Carl Gustav Carus, TU Dresden Dresden Germany

**Keywords:** copayment, expectation, robotic‐assisted surgery, urology, willingness to pay

## Abstract

**Objectives:**

To investigate the expectation of and copayment willingness for robotic‐assisted surgery (RAS) within a German cohort.

**Patients and methods:**

A cross‐sectional survey at a university hospital's open house gathered responses from 273 participants before and after RAS exhibition. The questionnaire assessed demographics, pre‐existing knowledge about and expectation of RAS as well as copayment willingness before and after live presentation of a multiport system. Statistical analyses, including Wilcoxon test, comparison of proportions test and multivariate logistic regression, identified key associations.

**Results:**

54.1% of participants assumed that RAS offers some advantages but is not additionally reimbursed. Confidence in robotic surgery significantly increased (*p* = 0.004) after RAS exhibition. Additionally, there was a significant shift (*p* < 0.0001) to accept postponement of surgery for RAS. Regarding copayment willingness, the median amount was €500 (95% confidence interval [95% CI] €0–3 000) before and €1 000 (95% CI €0–10 000) after RAS exhibition. Higher age (odds ratio 2.18; *p* = 0.0079) and net income >€2 500 were the only independent predictors for copayment amounts ≥€1 000.

**Conclusion:**

This study reveals that enabling the public to gain insight into RAS might lead to a better understanding of both the advances and limitations of this modern technology. In principle, the public accepts within limits postponement of surgery to receive RAS. To a certain extent, there is also a willingness for copayment to receive RAS, but the risk of persuasion by presenting innovative minimally invasive surgical approaches must be carefully taken into account.

## 1 INTRODUCTION

Robotic‐assisted surgery (RAS) has transformed modern urological practice and is now considered one of the most significant technological advances in minimally invasive surgery. Since the introduction of robotic platforms in the early 2000s, RAS has become the preferred surgical approach for several complex urological operations, especially when reconstruction is required.^1^, ^2^, ^3^, ^4^, ^5^ Even though this technology is perceived in everyday urological practice to result in fewer perioperative complications, reduced pain medication requirements and shorter hospital stays, prospective randomized studies that clearly demonstrate these benefits are often lacking.^6^, ^7^, ^8^ As healthcare systems increasingly emphasize value‐based care, balancing clinical benefits with economic sustainability has become a major priority. Consequently, statutory health insurers in Germany thus far do not provide additional reimbursement for the use of an RAS system in urology. This places additional pressure on hospitals, particularly given the current situation of tight budgets in the healthcare system. To date, most urology clinics in Germany do not require patients to pay additional fees for RAS. However, it is unclear whether this situation will remain the same or whether patient copayment will be standard in the future.

Especially when copayments are required in the medical field, patients should be adequately informed about technical specifications and potential benefits. Interestingly, a study by Hirtsiefer et al. demonstrated strong public support for RAS.^9^ However, prevalent misconceptions as well as willingness to pay for RAS have not yet been thoroughly investigated. Thus, the aim of the current study was to investigate the expectations of and willingness to pay for RAS before and after presentation of a multiport system and hands‐on training to the general public.

## 2 PATIENTS AND METHODS

In this single‐centre descriptive study, a paper‐based 16‐item survey was handed out to adult visitors of an open house event of the urologic department at a German university hospital. Open house was embedded in a city‐wide event propagating science to the general public. Participants were handed out first a nine‐item survey before entering the RAS exhibition to test their pre‐existing expectations, knowledge of costs and willingness for copayment to receive RAS. The RAS exhibition was divided into two parts. First, educational posters including robotic development, workflow of robotic surgery in daily clinical practice, studies on training in robotic surgery and its future perspectives were presented and critically discussed by medical staff members. This was followed by a live presentation of a multiport robotic system with hands‐on training. Afterward participants were asked to answer a seven‐item survey. The study was conducted in ethical compliance with the Declaration of Helsinki. The survey was conducted among members of the general public attending an open‐house presentation on RAS. Participation was voluntary and anonymous. Ethical approval was not required for this study. Under § 15 (1) of the Professional Code of the Saxon Chamber of Physicians (Berufsordnung der Sächsischen Landesärztekammer, as amended 4 December 2024, in force since 1 January 2025), review by an ethics committee is mandatory only for research projects that interfere with the physical or psychological integrity of a person or that use human biological material or data attributable to an identifiable individual. The present study was a fully anonymous questionnaire survey involving no intervention and no collection of identifiable or re‐identifiable data; no addresses, contact details or response codes were recorded. It did not constitute a clinical trial under the German Medicinal Products Act, the EU Clinical Trial Regulation or the Medical Devices Regulation.^10^ Participation was voluntary and could be declined without any disadvantage; participants were informed in writing about the scientific use of their responses and gave implied consent by returning the questionnaire. The questionnaire (see Appendix S1, translated to English) was in German language.

The survey began with biographical questions on age, gender, educational status (secondary, intermediate or high school or college), professional status (healthcare or technical/skilled trade or other), type of health insurance (statutory vs. private) and experience with previous robotic surgery. The first part of the survey included nine items: familiarity with robotic surgery, trust in robotic surgery, impact on outcome between surgeon and robot, reasonable postponement for robotic surgery, pro robotic arguments (multiple answers possible), limitation of RAS compared to open surgery (multiple answers possible), suggested price of a robotic system, reimbursement by statutory health system and personal copayment amount. The second seven‐item survey addressed the net income as well as the aforementioned six questions of trust in robotic surgery, impact on outcomes between surgeon and robot, reasonable postponement for robotic surgery, pro‐robotic arguments, limitations of robotic system compared to open surgery and willingness for copayment.

The Wilcoxon test, the Mann–Whitney test and the comparison of proportions test were used for univariate comparisons of questionnaire results before and after RAS system presentation. A multivariate logistic regression analysis (backward) was performed to define independent predictive variables for willingness to pay for RAS, before and after the RAS exhibition. Age (≥41 years vs. <41 years), gender (female vs. male), educational status (secondary and intermediate school vs. high school or college), professional status (healthcare and technical/skilled trade vs. other), type of health insurance (statutory vs. private) and net income (>€2 500 vs. <€2 500) were explored as potential predictors. MedCalc (MedCalc Software, Ostend, Belgium) was used for statistical analysis. This manuscript was written in accordance with STROBE (see Appendix S2).

## 3 RESULTS

273 participants filled out questionnaires in this first German single‐center study on robotic expectations of and willingness to pay for RAS.

### 3.1 Participants characteristics

The median age was 41 years (95% confidence interval [95% CI] 16–75 years). Sex was well balanced among participants (53.8% female vs. 45.9% male). One participant identified as ‘other’ for gender (0.3%). Regarding educational status, most participants achieved high school and/or college (73.6%), followed by intermediate school (24.6%) and secondary school (1.8%). According to professional status, 31.1% were healthcare professionals and 25.6% in technical or skilled trades, while 39.2% stated they work outside these professions. 83.1% had statutory health insurance and 16.9% private health insurance. Net income was <€1 000, €1 000–2 500, €2 500–5 000, >€5 000 or not answered in 7%, 32%, 34%, 7% or 21% of participants. Participant characteristics are shown in Table 1.

**TABLE 1:** Participants characteristics.

Age (years)	
Median (95% CI)	41 (16–75)
Sex (*n*, %)	
Female	147 (53.8)
Male	125 (45.9)
Other	1 (0.3)
Educational status (*n*, %)	
Secondary school	5 (1.8)
Intermediate school	67 (24.6)
High school	51 (18.7)
College	150 (54.9)
Professional status (*n*, %)	
Healthcare	85 (31.1)
Technical/skilled trade	70 (25.6)
Other	107 (39.2)
n.a.	11 (4.1)
Type of health insurance (*n*, %)	
Statutory	227 (83.1)
Private	46 (16.9)
Net income (*n*, %)	
<€1 000	19 (7)
€1 000–2 500	86 (32)
€2 500–5 000	93 (34)
>€5 000	19 (7)
n.a.	56 (21)

Abbreviations: CI, confidence interval; n.a., not answered; RAS, robotic‐assisted surgery.

### 3.2 Pre‐existing knowledge about RAS

Previous surgery was reported in 69.6% of participants, of whom 10.5% were treated using robotic approach. Regarding familiarity with RAS, most of the participants stated to be not at all familiar (35.9%), followed by slightly (32.6%) and somewhat (25.2%) familiar. The majority of participants expected a price tag for RAS systems >€1 000 000 (75.8%). Regarding a possible reimbursement of RAS costs by statutory health insurance, 54.1% of participants assumed that RAS offers some advantages but is not additionally reimbursed. In contrast, 39.9% of participants assumed that RAS offers advantages and is reimbursed. The minority expected RAS to be not advantageous at all (6%). Data are shown in Table 2.

**TABLE 2:** Pre‐existing knowledge about RAS.

Experience to previous surgery (*n*, %)	
Yes, including at least one RAS	20 (7.3)
Yes, but not RAS	170 (62.3)
No, I have never had surgery	83 (30.4)
Familiarity with robotic surgery (*n*, %)	
Very familiar	17 (6.3)
Somewhat familiar	69 (25.2)
Slightly familiar	89 (32.6)
Not familiar at all	98 (35.9)
Price range of RAS system (*n*, %)	
€100 000–499 000	14 (5.2)
€500 000–1 000 000	52 (19.0)
€1 000 000–1 999 000	105 (38.5)
€2 000 000 or more	102 (37.3)
True statement regarding reimbursement of RAS by statutory healthcare (*n*, %)	
Offers no advantages, is not additionally reimbursed	8 (3.0)
Some advantages, is not additionally reimbursed	145 (54.1)
Offers some advantages, is additionally reimbursed	107 (39.9)
Offers no advantages, is additionally reimbursed	8 (3.0)

Abbreviation: RAS, robotic‐assisted surgery.

### 3.3 Expectation and willingness to pay

Six items of the survey were evaluated before and after RAS exhibition. Regarding confidence in RAS, the majority of participants assumed before and after the RAS exhibition that surgeon and the RAS had the same impact on surgical outcomes (36.8% and 36.0%, respectively). Interestingly, there was a significant trend (*p* = 0.004) pro RAS after visiting the exhibition (Figure 1A). When participants were asked before RAS exhibition if they would postpone kidney tumour surgery for RAS, 50.2% and 37.1% and 7.3% and 5.4% of participants were willing to wait 2, 4, 6 or 8 weeks. After RAS exhibition, there was a significant shift (*p* < 0.0001) to postpone surgery for robotic approach (32.7%, 38.3%, 18.2% and 10.8% to wait for 2, 4, 6 or 8 weeks). Only one patient refused to wait for RAS. Data are shown in Figure 1B.

**FIGURE 1:**
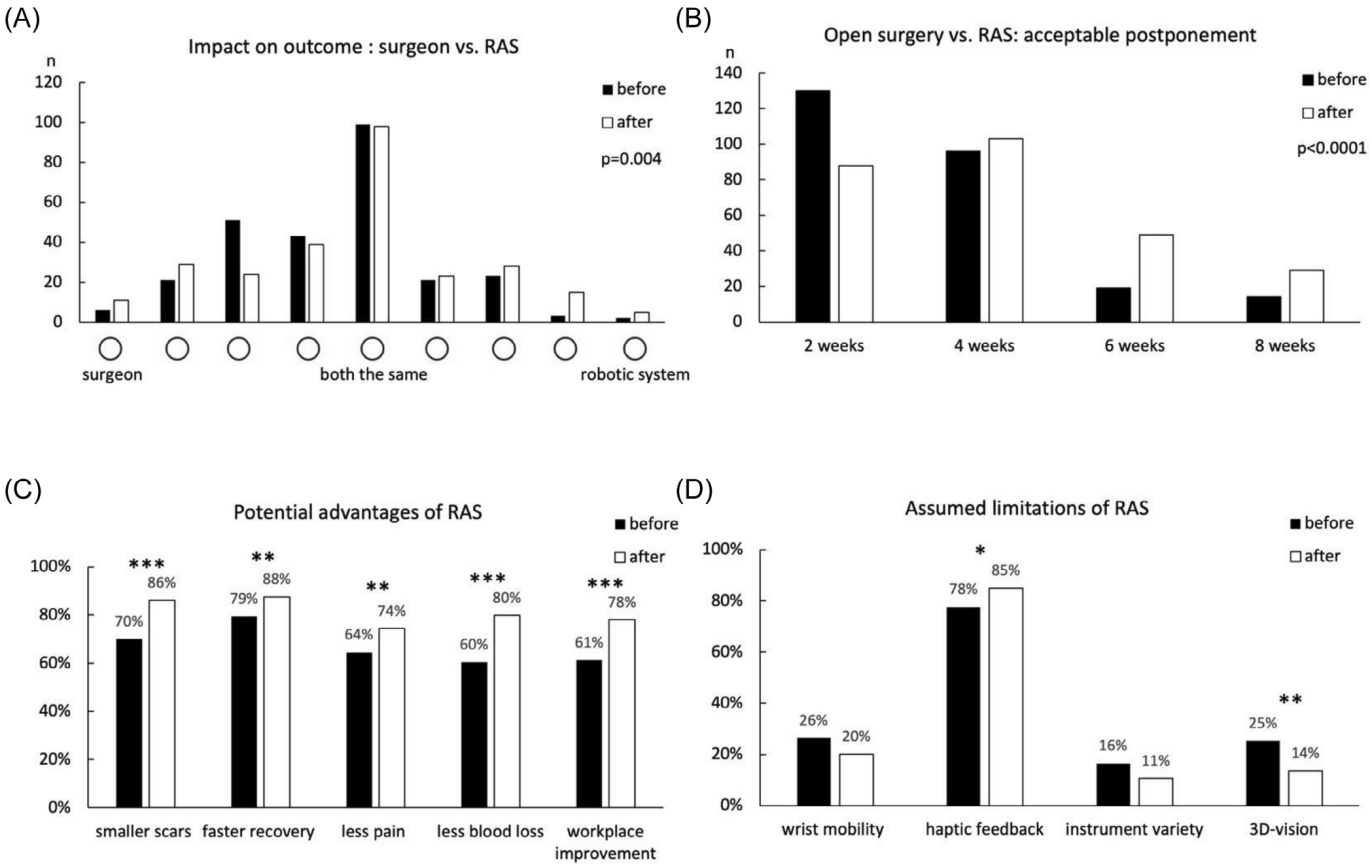
Expectations on RAS *before* and *after* visiting the exhibition. (A) Impact on outcome between surgeon and RAS, (B) postponement of kidney tumour surgery for RAS, (C) potential advantages of RAS (multiple answers possible) and (D) assumed limitations of RAS (multiple answers possible). 3D, three‐dimensional; RAS, robot‐assisted surgery; *0.05 < *p* > 0.01; **0.01 < *p* > 0.001; ***p < 0.001.

The expectations regarding potential advantages of RAS significantly increased after exhibition (Figure 1C). Before RAS exhibition, the most commonly assumed limitations of RAS were impaired haptic feedback (78%), followed by limited wrist mobility (26%), missing 3D‐vision (25%) and small variety of instruments (16%). After RAS exhibition, impaired haptic feedback as a limitation was significantly increased to 85% (*p* = 0.046), while missing 3D‐vision was significantly less stated (14%; *p* = 0.0017). Data are shown in Figure 1D.

The willingness to make copayment amounts for RAS was very heterogeneous before the RAS exhibition, with 12%, 15%, 25%, 13%, 19%, 5% and 10% willing to pay €0, €250, €500, €750, €1 000, €2 000 and €3 000. The median copayment amount was €500 (95% CI €0–3 000). After RAS exhibition, the willingness for copayment significantly (*p* < 0.0001) increased to a median of €1 000 (95% CI €0–10 000). Post exhibition, the rejection of copayment dropped to 8%.

In multivariate analyses addressing the willingness to copay for RAS before taking part in the exhibition, only higher age of participants (≥41 years; OR 2.16; *p* = 0.0195) was identified as an independent predictor for personal copayment ≥€500 (Table 3A). After RAS system presentation, higher age (OR 2.18; *p* = 0.0079) as well as a net income >€2 500 (OR 1.79; *p* = 0.0472) were identified as independent predictors of willingness for copayment ≥€1 000 (Table 3B). Gender, educational status, professional status and type of health insurance were not independent predictors for copayment willingness before or after the RAS exhibition. Participants not disclosing income were excluded from multivariate analysis. Of note, there was no significant difference in the median copayment amount between disclosers and non‐disclosers before RAS presentation (€500 vs. €500; *p* = 0.93) as well as after presentation (€1 000 vs. €900; *p* = 0.99).

**TABLE 3A:** Multivariate logistic regression analysis (backward) of independent variables *before RAS exhibition* for willingness of copayment ≥€500 (*n* = 205).

	Odds ratio	95% CI	Coefficient	SE	*p*‐value
Age ≥41 years vs. <41 years	2.1609	1.1319–4.1256	0.77054	0.32993	0.0195

*Note*: Gender (female vs. male), educational status (secondary and intermediate school vs. high school or college), professional status (healthcare and technical/skilled trade vs. other), type of health insurance (statutory vs. private) and net income (>€2 500 vs. <€2 500) were not independent variables.

**TABLE 3B:** Multivariate logistic regression analysis (backward) of independent variables *after RAS exhibition* for willingness of copayment ≥€1 000 (*n* = 204).

	Odds ratio	95% CI	Coefficient	SE	*p*‐value
Age ≥41 years vs. <41 years	2.1802	1.2267–3.8750	0.77943	0.29343	0.0079
Net income >€2 500 vs. <€2 500	1.7902	1.0073–3.18116	0.58234	0.29339	0.0472

*Note*: Gender (female vs. male), educational status (secondary and intermediate school vs. high school or college), professional status (healthcare and technical/skilled trade vs. other) and type of health insurance (statutory vs. private) were not independent variables.

### 3.4 Expectation and willingness to pay in participants with somewhat/very familiarity with robotic surgery

To investigate if familiarity with robotic surgery is impacting willingness to pay as well as postponement of renal cancer surgery, we analysed the subgroup that stated to be somewhat/very familiar with robotic surgery (*n* = 86). Interestingly, these participants had a significantly higher willingness to pay before RAS presentation compared to participants slightly/not familiar with robotic surgery (€750 [95% CI €609–1 000] vs. €500 [95% CI €500–500]; *p* = 0.02). After RAS presentation, there was no significant difference in willingness to pay between both groups (€1 000 [95% CI €749–1 000] vs. 1 000 [95% CI €500–1 000]; *p* = 0.58). Regarding postponement of renal cancer surgery, there was still a significant shift (*p* = 0.03) to postponement of surgery for robotic approach in participants being somewhat/very familiar with robotic surgery (Figure 2).

**FIGURE 2:**
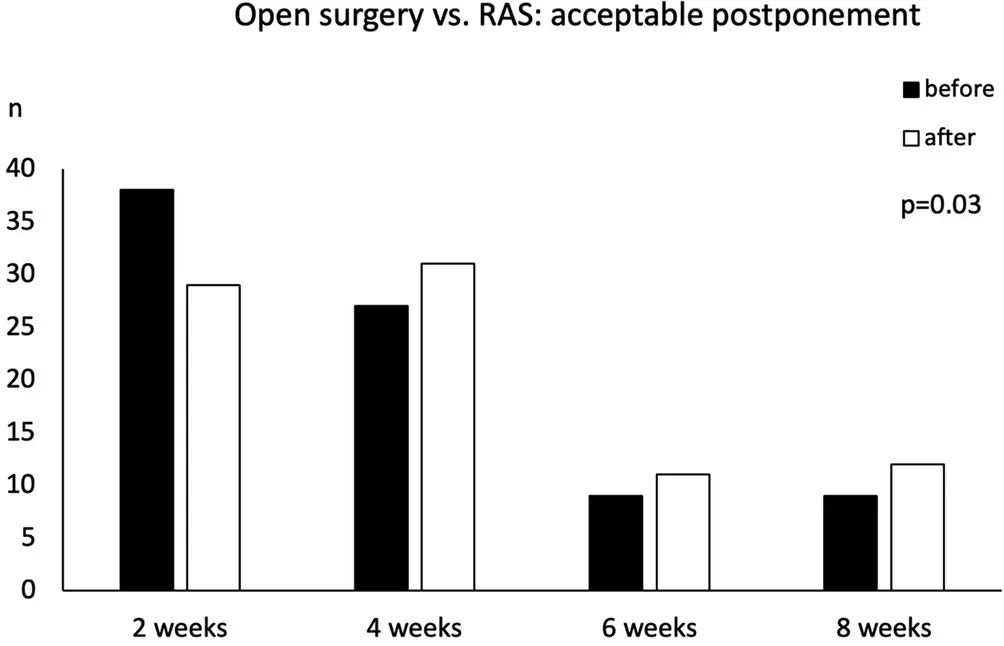
Postponement of kidney tumour surgery for RAS *before* and *after* visiting the exhibition in participants with somewhat/very familiarity in robotic surgery. RAS, robot‐assisted surgery.

## 4 DISCUSSION

The aim of our study was to investigate the expectation of and willingness to pay for robotic‐assisted surgery before and after RAS live presentation and hands‐on training for the general public. Here, we show that enabling the general public to get insight in RAS leads to improved understanding of this technology and is associated with a higher willingness for copayment, especially after visiting a RAS exhibition with hands‐on training.

The general public is often unaware of the technical details underlying RAS, leading to misconceptions in four of 10 participants of scientific open house events.^9^ Nevertheless, seven out of 10 would still favour RAS over conventional surgery. Our findings support that the public is not well informed about RAS, as one in three participants at our open house event were not familiar at all and only one in four participants stated they were somewhat familiar with this modern technology. Interestingly, live RAS presentation with hands‐on training significantly increased participants expectations regarding advances and limitations of this modern technology (Figure 1C,D). Moreover, confidence in the robotic system significantly increased (*p* = 0.004) after visiting the RAS exhibition (Figure 1A). Our findings are supported by Fumagalli et al., who demonstrated that live presentation of robot‐assisted spine surgery led to a significantly higher confidence in RAS.^11^

From an ethical standpoint, the postponement of oncological surgeries is often viewed critically from the patient's and doctor's perspective. Especially patients usually lack the clinical knowledge needed to assess the severity of their condition and surgery as soon as possible is often requested. Delay of surgery typically leads to anxiety, anger, frustration and willingness to change surgeons.^12^ However, the need to postpone RAS in daily clinical practice can certainly happen, especially when robotic resources are not widely available. Therefore, we addressed this context in our questionnaire with an example of renal cell carcinoma, where enough evidence is available that temporary postponement of surgery is medically justifiable in most cases.^13^, ^14^ For example, Shiff et al. were able to show in a large population of 1769 renal cell carcinoma patients that surgical wait times up to 24 weeks were not associated with adverse oncologic outcomes after 2 years of follow‐up. In our study, the acceptance of surgical postponement to receive RAS, which was limited to a maximum of 8 weeks, significantly increased (*p* < 0.0001) when participants attended the RAS exhibition (Figure 1B). Interestingly, when focusing solely on participants with somewhat/very familiarity with robotic surgery, there was still a significant shift to postpone surgery to receive robotic approach (*p* = 0.03). This might be explained by a limited durability of knowledge regarding robotic surgery even in citizens that assume to possess relevant knowledge about RAS.

Reimbursement of costs related to RAS is a sensitive topic in the German healthcare system. Typically, hospitals receive the usual DRG (Diagnosis‐Related Group) payment for the surgery, regardless of whether it is performed open, laparoscopically or robotically. The additional costs of the RAS system (capital costs, maintenance, instruments) are generally not separately reimbursed.^15^ However, this information is not known to everyone in the general public. In our study, only every second participant assumed that RAS offers some advantages but is not reimbursed by statutory healthcare.

Overall, the healthcare system in Germany faces a major challenge due to increasing costs, particularly in the inpatient sector.^16^ Given the expected cost‐cutting measures, patients must also consider the possibility of future copayments for specialized medical services.

In our study, we examined for the first time the general public's willingness to pay for robotic surgery in Germany, both before and after a live demonstration of a robotic multiport system. We were able to show that to a certain extent, there is a willingness for copayment to receive RAS, with a median amount of €500 before the RAS exhibition presentation and a median amount of €1 000 thereafter. Only 8% of the participants could not imagine copayment for RAS even after the exhibition. While higher age (≥41 years) was an independent predictor for higher amount of copayment before and after RAS exhibition, a net income >€2 500 was an additional independent predictor for a higher amount of copayment after RAS exhibition. This finding seems to be reasonable as citizens in Western countries have the greatest financial capacity to absorb additional costs or make trade‐offs starting in of the fourth decade of life.^17^

Of note, in participants with somewhat/very familiarity with robotic surgery, there was a significantly higher willingness to pay before RAS presentation compared to participants slightly/not familiar with robotic surgery (*p* = 0.02). After RAS presentation, there was no significant difference in willingness to pay between both groups (*p* = 0.58). This observation may be explained by the fact that patients that are somewhat/very familiar with RAS may have already copayed for RAS or have a better understanding for this complex surgical technique.

## 5 STRENGTHS AND LIMITATIONS

Before we discuss the strengths and limitations of our research project, it is important to us to state that this manuscript was not written with the intention to promote robotic surgery or to require patients to make copayments for surgical procedures in the future. We are aware that presenting novel surgical techniques to the general public carries the risk of focusing just one‐sided on the advantages and neglecting the limitations. For this reason, we have included an objective educational poster presentation on robotic surgery in our robotic exhibition. Nevertheless, particularly in urology, we should not ignore the fact that the surgical trend is clearly moving towards RAS. And it is well known that, at least in Germany, that this is associated with a significant additional financial burden for healthcare providers.

The strength of our study lies in its prospective design and the evaluation of RAS‐specific questions both before and after the presentation of a multiport system. This allows for a direct assessment of the technology presentation's impact on the participants. In addition, we determined an amount of money for copayment willingness for the first time. Even though the topics of finances and copayments are often sidestepped in academic discussions in the healthcare sector, they will play an increasingly important role in the future.

However, our study also has several limitations that reduce the validity of the data. These include a bias by prespecified participant population with the absence of a naive baseline control group, the single‐centre setting, limited data regarding net income and the unknown durability of the observed effects. Therefore, we want to go in more detail regarding some of these limitations. In fact, 21% of the participants refused to provide anonymous information regarding their net income. On the other hand, setting the cut‐off at €2 500 for income classification in our study corresponds fairly closely to the average net salary of a single person in Germany.^18^ Moreover, the median amount of copayment did not significantly differ between disclosers and non‐disclosers before as well as after presentation.

Another limitation is the fact that our cohort does not represent the general population, as the distribution among professions shows an over‐representation of healthcare professionals (31.1% in our cohort vs. 18%) in Germany.^19^ This limitation cannot be eliminated, and it is to be expected that participants with a healthcare background will be—due to their inherent interest in science—frequently over‐represented at scientific open‐house events. It must also be said that 273 visitors at an open house event at a German university hospital should provide profound insights about expectations and willingness to pay for RAS. A certain limitation is also evident when comparing the responses to the question about willingness to pay before and after RAS presentation (Q9 vs. Q15). However, in our understanding, it was important for the participants to be given a categorial price range before RAS presentation with the opportunity to define the individual copayment amount afterwards.

## 6 CONCLUSION

Our study reveals that enabling the public to gain insight into robotic surgery might lead to a better understanding of both the advances and limitations of this modern technology. In case of robotic shortage, the public accepts within limits the postponement of surgery to receive RAS. To a certain extent, there is also a willingness of copayment for RAS, especially among participants who were able to discuss all pros and cons of this topic with medical experts as part of a live presentation. Important to note, the risk of persuasion by presenting innovative minimally invasive surgical approaches must be carefully taken into account.

## AUTHOR CONTRIBUTIONS

*Study concept and design*: Christian Thomas, Martin Baunacke and Mandy Ehrt. *Acquisition of data*: Maximilian Winkelkotte, Emma Peklo, Mario Hallaschek and Katharina Sonntag. *Analysis and interpretation of data*: Christian Thomas and Martin Baunacke. *Drafting of the manuscript*: Christian Thomas. *Critical revision of the manuscript for important intellectual content*: Christian Thomas, Martin Baunacke and Benedikt Hoeh. *Statistical analysis*: Christian Thomas and Benedikt Hoeh. *Administrative, technical or material support*: Mandy Ehrt and Benedikt Hoeh. *Supervision*: Christian Thomas.

## ETHICS STATEMENT

The study was conducted in compliance with the Declaration of Helsinki. Consent to participate in the survey anonymously was precondition to visiting the exhibition on RAS. Written consent was not required as no medical information was obtained.

## CONFLICT OF INTEREST STATEMENT

The authors declare to have no conflicts of interest.

## Supporting information


**Appendix S1.** Supporting Information.

**Appendix S2.** Supporting Information.

## Data Availability

The data that support the findings of this study are available on request from the corresponding author. The data are not publicly available due to privacy or ethical restrictions.
