# Supplementary material for: Expectations of and willingness to pay for robotic‐assisted surgery: A prospective analysis before and after live presentation to the public
Source: BJUI Compass. 2026 Nov 13;7(11):e70304. doi: 10.1002/bco2.70304 (PMC13768203; doi:10.1002/bco2.70304)
Supplement: Supplementary file 1 [file BCO2-7-e70304-s001.pdf]

Long Night of Science 2026

Robot-Assisted Surgery and Health Economic Aspects

We are interested in how people think about robot-assisted surgery.

In particular, we would like to find out whether you are familiar with the technical capabilities and the associated costs of the robotic system presented here.

Our questionnaire is based on an evaluation before and after the demonstration of the system. Please answer the questions accordingly.

All responses are anonymous.

The collected data will be used for scientific research and may also be published.

Demographic Information

Age: _______

Gender:
☐ Male
☐ Female
☐ Diverse / Non-binary

Highest level of education completed:
☐ Secondary school
☐ Intermediate school
☐ High school
☐ College

Do you currently work (or have you worked) in:
☐ Healthcare
☐ Technical or skilled trades
☐ Other field

Health insurance:
☐ Statutory
☐ Private

Have you ever had surgery?
☐ Yes, including at least one robot-assisted procedure

☐ Yes, but not a robot-assisted procedure
☐ No, I have never had surgery

Before the Demonstration – part 1

1. How familiar are you with robot-assisted surgery?

○ Very familiar

○ Somewhat familiar

○ Slightly familiar

○ Not familiar at all

---

2. How much do you trust the safety of robot-assisted surgery compared with conventional surgery?

○ Very much

○ A lot

○ Moderately

○ A little

○ Not at all

---

3. The outcome of an operation depends on the interaction between the surgeon and the robotic system.

Please mark the circle that best represents what you believe is the appropriate balance between the surgeon and the robotic system.

○ ○ ○ ○ ○ ○ ○ ○ ○

surgeon both the same robotic system

---

4. If you needed surgery for a kidney tumor and could either undergo immediate open surgery or wait for robot-assisted surgery, what is the longest you would be willing to wait for the robotic procedure?

○ 2 weeks

○ 4 weeks

○ 6 weeks

○ 8 weeks

---

5. Which potential advantages of robot-assisted surgery do you find most appealing? *(Multiple answers possible.)*

☐ Smaller scars

☐ Faster recovery

☐ Less pain

☐ Less blood loss

☐ Improved working conditions for the surgeon

---

6. Compared with open surgery, what do you believe cannot be reproduced—or is reproduced less effectively—with robot-assisted surgery? *(Multiple answers possible.)*

☐ Wrist mobility

☐ Sense of touch (haptic feedback)

☐ Variety of instruments

☐ Three-dimensional visualization

---

7. What do you think is the purchase price of a standard surgical robotic system?

○ €100,000–499,000

○ €500,000–1,000,000

○ €1,000,000–1,999,000

○ €2,000,000 or more

---

8. Which statement do you believe is true regarding reimbursement for robot-assisted urological surgery for patients covered by statutory health insurance in Germany?

○ It offers no advantages and therefore is not reimbursed additionally by health insurance.

○ It offers some advantages but is still not reimbursed additionally by health insurance.

○ It offers some advantages and is reimbursed additionally by health insurance.

○ It offers no advantages but is nevertheless reimbursed additionally by health insurance.

---

9. Would you be willing to pay the following additional amounts to have your surgery performed using robot-assisted technology?

Additional Payment	Yes	No
€250	☐	☐
€500	☐	☐
€750	☐	☐
€1,000	☐	☐
€2,000	☐	☐
€3,000	☐	☐

After the Demonstration – part 2

10. How much do you trust the safety of robot-assisted surgery compared with conventional surgery?

○ Very much

○ A lot

○ Moderately

○ A little

○ Not at all

---

11. The outcome of an operation depends on the interaction between the surgeon and the robotic system.

Please mark the circle that best represents what you believe is the appropriate balance between the surgeon and the robotic system.

○ ○ ○ ○ ○ ○ ○ ○ ○

surgeon both the same robotic system

---

12. If you needed surgery for a kidney tumor and could either undergo immediate open surgery or wait for robot-assisted surgery, what is the longest you would be willing to wait for the robotic procedure?

○ 2 weeks

○ 4 weeks

○ 6 weeks

○ 8 weeks

---

13. Which potential advantages of robot-assisted surgery do you find most appealing? *(Multiple answers possible.)*

☐ Smaller scars

☐ Faster recovery

☐ Less pain

☐ Less blood loss

☐ Improved working conditions for the surgeon

---

14. Compared with open surgery, what do you believe cannot be reproduced with robot-assisted surgery? *(Multiple answers possible.)*

☐ Wrist mobility

☐ Sense of touch (haptic feedback)

☐ Variety of instruments

☐ Three-dimensional visualization

---

15. Assuming robot-assisted surgery would require a copayment amount, what is the maximum amount you would personally be willing to pay to have your surgery (for example, kidney tumor removal) performed robotically?

Amount in euros: ___________________ €

---

16. Please indicate your monthly net income.

☐ Less than €1,000

☐ €1,000–2,500

☐ €2,500–5,000

☐ More than €5,000

☐ Prefer not to answer
